# Smart Nanotherapeutics and Lung Cancer

**DOI:** 10.3390/pharmaceutics13111972

**Published:** 2021-11-20

**Authors:** Mohammad Doroudian, Mohammad H. Azhdari, Nima Goodarzi, David O’Sullivan, Seamas C. Donnelly

**Affiliations:** 1 School of Medicine, Trinity Biomedical Sciences Institute, Trinity College, Dublin 2, Ireland; mdoroudi@tcd.ie (M.D.); Osulld16@tcd.ie (D.O.); 2 Department of Cell and Molecular Sciences, Faculty of Biological Sciences, Kharazmi University, Tehran 15719-14911, Iran; azhdari.mh99@gmail.com (M.H.A.); nima.goodarzi99@gmail.com (N.G.); 3 Department of Clinical Medicine, Trinity Centre for Health Sciences, Tallaght University Hospital, Tallaght, Dublin 24, Ireland

**Keywords:** nanomedicine, smart nanoparticles, nanotherapeutics, lung cancer, nanodrug delivery, targeted drug delivery, nanotechnology, nanochemotherapeutics, combination therapy

## Abstract

Lung cancer is a significant health problem worldwide. Unfortunately, current therapeutic strategies lack a sufficient level of specificity and can harm adjacent healthy cells. Consequently, to address the clinical need, novel approaches to improve treatment efficiency with minimal side effects are required. Nanotechnology can substantially contribute to the generation of differentiated products and improve patient outcomes. Evidence from previous research suggests that nanotechnology-based drug delivery systems could provide a promising platform for the targeted delivery of traditional chemotherapeutic drugs and novel small molecule therapeutic agents to treat lung cancer cells more effectively. This has also been found to improve the therapeutic index and reduce the required drug dose. Nanodrug delivery systems also provide precise control over drug release, resulting in reduced toxic side effects, controlled biodistribution, and accelerated effects or responses. This review highlights the most advanced and novel nanotechnology-based strategies, including targeted nanodrug delivery systems, stimuli-responsive nanoparticles, and bio-nanocarriers, which have recently been employed in preclinical and clinical investigations to overcome the current challenges in lung cancer treatments.

## 1. Introduction

Lung cancer creates a significant global disease burden and is the most common cause (accounting for 18%) of cancer-related deaths worldwide [1]. Moreover, lung cancer is the most frequent second primary malignancy, which is when a new cancer is identified in a person that is unrelated to an earlier cancer diagnosis [2]. There are two main types of lung cancer: small cell lung cancer and nonsmall cell lung cancer (NSCLC). Nearly 85% of patients with lung cancer suffer from NSCLC; this type is categorized into three subtypes: (i) adenocarcinoma, (ii) squamous cell carcinoma, and (iii) large cell carcinoma. The main cause of lung cancer is tobacco smoking which is responsible for around 80% of cases in the United States and other countries with high smoker ratios. Genetics, pollution, environmental radon, second-hand smoke, and asbestos are other factors that may cause lung cancer. Treatment options depend on many factors (e.g., cancer stage and the side effect of treatment) and mainly include surgery, chemotherapy, radiotherapy, immunotherapy, and targeted therapy [3,4]. Therapeutic interventions with anticancer drugs have seen large scale developments in their use in recent years, yet problems and challenges in the treatment of lung cancer still exist. While 10-year survival rates for numerous cancers have increased significantly in the last 30 years (e.g., prostate cancer from 25–84%), similar improvements have not been seen with lung cancer (5-year survival below 20%), particularly in metastatic disease [3,5]. An impressive body of evidence supports the concept that novel approaches are required to improve the efficiency and specificity of current lung cancer treatments [6,7,8].

**Figure 1:**
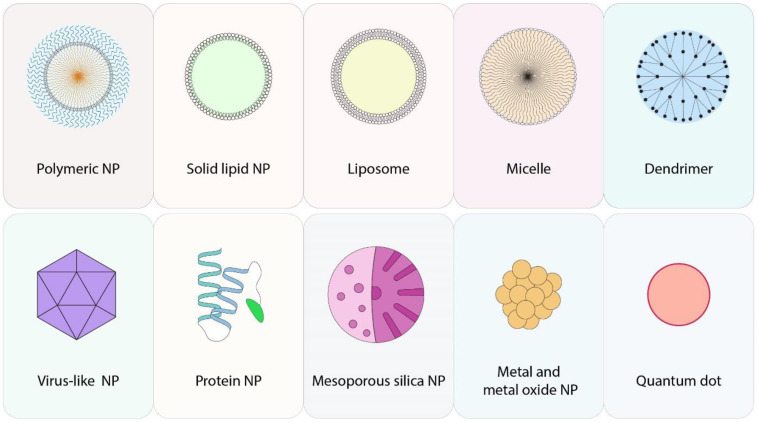
Various types of nanoparticles (NPs). Polymeric NPs, with the size of 1 to 10,000 nm, consist of monomers and can contain or entrap drugs or specific contents; **solid lipid NPs** are solid core spherical particles made from lipids with high structural stability; **liposomes** are artificial vesicles with at least one lipid layer with the size of 20 to 250 nm; a **micelle** is a colloidal particle consisting of the aggregation of amphiphilic molecules; ranging from 1.5 to 10 nm, **Dendrimers** are NPs with radially symmetric branches; in **virus-like NPs**, capsids of viruses are used as a rigid container for loading therapeutic drugs; **protein NPs** are composed of a therapeutic agent conjugated into a protein. Abraxane is a well-known example of protein NPs; **mesoporous silica NPs** are silica particles containing pores with the size of 2 to 50 nm; different metals can be used in the structure of NPs, typically in the core of NP, to improve the efficacy of drug delivery. **Metal and metal oxide NPs** are sensitive to many stimuli such as magnetic fields and radiation; **quantum dots** are artificial semiconductor nanocrystals with extensive application. In nanomedicine, quantum dots can be used as a tool for drug delivery.

Nanotechnology-based therapy has been shown to be a promising strategy by which cancer cells can be treated without harming healthy cells [6]. This has consequently become a fast-growing field with numerous medical and therapeutic applications. As one of the widely investigated applications for nanotechnology, nano-based drug delivery systems offer promise for the treatment of lung cancer and various diseases [9,10]. Nanocarriers have become promising tools in cancer therapy due to their intrinsic ability to overcome the current challenges associated with traditional chemotherapy anticancer drugs, including poor specificity, high systemic toxicity, and low water solubility [11,12]. Various types of nanomaterials such as liposomes, solid lipid nanoparticles (SLNs), polymers, dendrimers, and metallic nanoparticles have been employed to increase the delivery of anticancer therapeutics to tumor sites without affecting healthy tissues (Figure 1) [13,14].

Doxil^®^ was the first nanoparticle-based drug delivery approved by the FDA in 1995, and it encapsulates doxorubicin (DOX) chemotherapeutic drugs in liposomal nanoparticles to improve the drug’s prolonged circulation time and toxicity profile [15]. A decade later, in 2005, the FDA approved another nano-based anticancer drug, nanoparticle albumin-bound paclitaxel (NAB-PTX), also known as Abraxane. Abraxane provides numerous advantages including better overall efficiency, a reduced hypersensitivity reaction, an increase in life years gained (LYG) and quality-adjusted life years gained (QALYG) when compared to paclitaxel (PTX). As a result of these promising outcomes, the FDA approved Abraxane for the treatment of patients with NSCLC in 2012 [16]. Biomolecules such as proteins, peptides, aptamers, DNA, and RNA can be loaded into nanoparticles (NPs) to increase the therapeutic efficiency [17,18,19,20]. Despite these enormous advances, one of the most significant challenges in cancer therapy is the failure to develop drugs with tumor specificity [21]. This obstacle in cancer treatment can be addressed by using smart nanodrug delivery systems. This novel approach improves the therapeutic index, reduces the required drug dose, and allows for the control of drug release at the desired site [7,22,23].

## 2. Smart Nanodrug Delivery Systems

**Figure 2:**
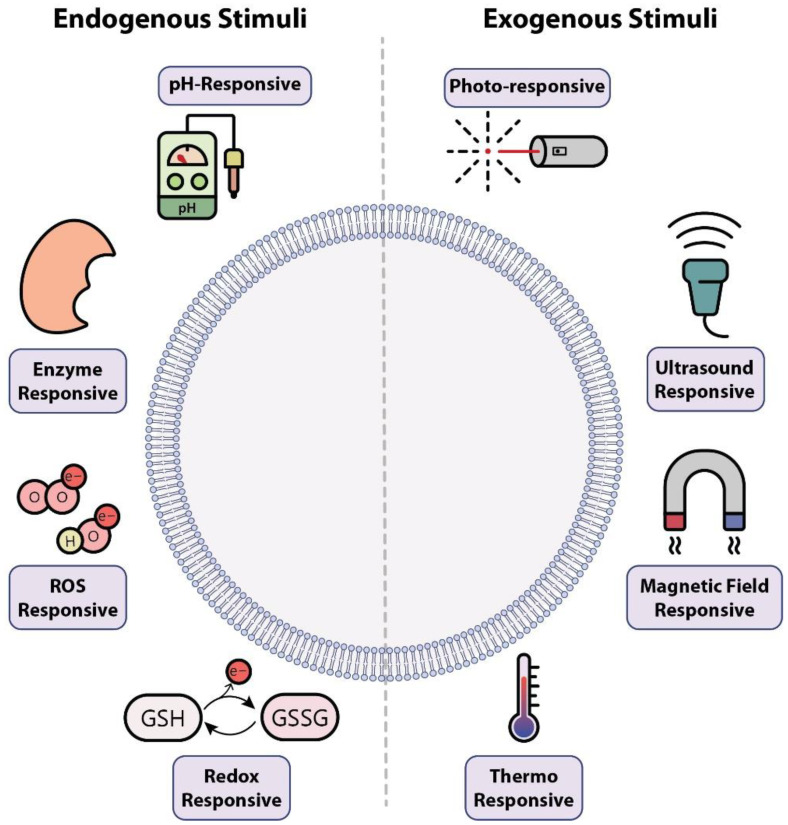
Endogenous and exogenous stimuli. One of the strategies in designing smart NPs is to make them sensitive to one or more stimuli. These stimuli could be endogenous (i.e., related to the intrinsic properties of the tumor microenvironment) or exogenous (i.e., stimuli that are applied from outside of the body). Well-known endogenous stimuli are pH (tumor microenvironments have lower pH than healthy tissue), overexpressed enzymes (some enzymes like matrix metalloproteinases are more expressed in tumor cells), reactive oxygen species (ROS), and redox agents (both ROS and redox agents are more concentrated in the tumor microenvironment). NPs can be designed to respond to exogenous stimuli such as light (photoresponsive), ultrasound, magnetic fields, and temperature (thermoresponsive).

Smart nanodrug delivery systems are new methods that could be used to help overcome the current challenges that exist in lung cancer treatments, such as drug resistance and lack of tumor specificity [6]. These promising strategies are categorized into two main groups: (i) targeted nanodrug delivery systems, and (ii) stimuli-responsive nanodrug delivery systems [7]. Targeted nanodrug delivery is a form of drug transfer that specifically delivers therapeutic agents to the desired action site to localize drug interactions with the diseased area. Consequently, this helps to evade adverse drugs effects, such as damage to healthy cells [6]. Stimuli-responsive nanodrug delivery systems represent a precisely controlled release profile for therapeutic agents [24]. They have been designed to release their cargo into specific tissues and, when confronted with unique stimulating factors, to achieve efficient drug delivery [25]. These stimulations can be divided into “endogenous” (e.g., pH, redox agents, and enzymes) and “exogenous” (e.g., light, ultrasound, and magnetic field) categories [26,27] (Figure 2).

### Targeting Strategy

To enhance the therapeutic effects of NPs, they should have the capacity to selectively deliver their cargo to tumor sites. The two main targeting approaches used in targeted nanodrug delivery systems are: (i) active targeting and (ii) passive targeting. In passive targeting, NPs accumulate in the tumor sites rather than in healthy tissues due to the enhanced permeability and retention (EPR) effect. Active targeting generally depends on targeting ligands attached to the surface of the NPs to bind to specific receptors or molecules on tumor cells [10,28].

The irregular formation of blood vessels and lack of normal vascular basement membrane structures in tumor sites causes an influx of NPs into these areas. This phenomenon is known as the EPR effect and it helps to facilitate nanoparticle accumulation in tumors, which is known as “passive targeting”. This process is based on nanoparticle physiochemical properties and intrinsic tumor features and leads to drug accumulation at the tumor site [25,29]. The properties of NPs, such as size, shape, and surface characteristics, affect the drug delivery efficiency through the EPR effect. Nanoparticles with sizes between 40 to 400 nm are favorable to remain longer time in circulation. Also, NPs with this range of size have more accumulation in tumor sites and have the appropriate size to bypass uptake by the liver and spleen and escape from renal clearance. For instance, Magnetic NPs such as iron oxide NPs are commonly used as nanodrug delivery systems [30,31], and modifying their size could change their biodistribution and accumulation in the liver and spleen. Designing NPs larger than 200 nm increases the risk of their degradation by spleen and kidney, whereas NPs smaller than 30 nm are more likely to return into the vessel from tumor tissue. Therefore, to avoid the removal of NPs from circulation, it is crucial to develop NPs with suitable sizes (between about 30 to 200 nm) [32]. The surface charge of the NPs is another factor that determines its durability in blood circulation and reduces their removal over the reticuloendothelial system (RES) [33,34]. In one study, PTX and DOX were loaded into nanostructured lipid carriers (PTX-DOX-NLC) and examined using multidrug-resistant NSCLC. PTX-DOX-NLC showed more anticancer activity than the combination of single drugs with or without NLCs, indicating that passive targeting has a high tumor-targeting capacity [35]. The lymphatic system plays an important role in the spread of cancer. Thus, targeting this system can decrease cancer cell spread and increase antitumor activity [36]. To address this issue, docetaxel (DTX)-loaded polyglutamic acid-polyethylene glycol (PGA-PEG) nanocapsules were developed and examined in a metastasizing orthotopic lung cancer model. The small size of the NPs (approximately 100 nm) lead to a significant accumulation in the lymphatic nodules, which provided a greater anticancer effect when compared to free DTX and successfully eliminated metastatic cells in the lymph nodes [37].

**Figure 3:**
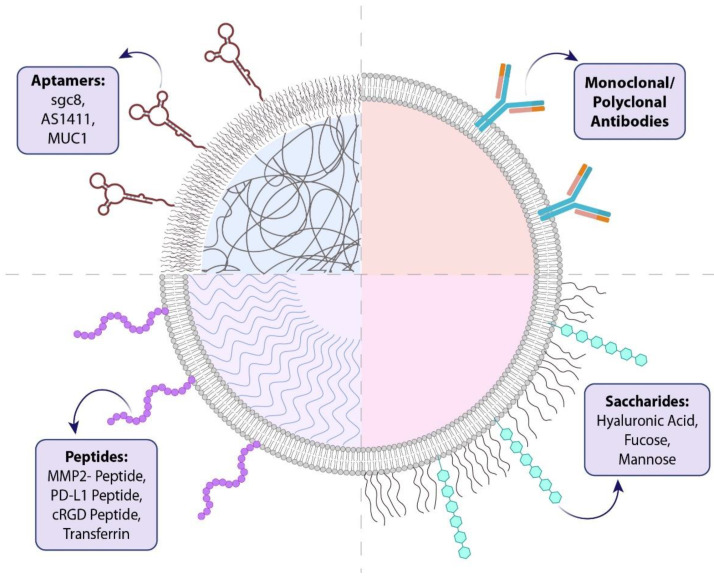
Different molecules that can be utilized for targeting nanoparticles. Different molecules can be employed to generate targeted nanodrug delivery systems for the treatment of lung cancer. These molecules are chosen according to overexpressed receptors or membrane proteins, or even their affinity for the whole tumor cell. Small peptides, saccharides, antibodies, and aptamers are the most common molecules utilized to target nanoparticles.

Active targeting increases the cellular uptake of NPs at tumor sites and improves drug delivery efficiency [38]. Targeted drug delivery can be achieved by functionalizing the NP surface to bind specifically to cancer cell receptors. This strategy reduces the required dose of the delivered drug, minimizes side effects, increases the concentration of the therapeutic agent at the desired site, and enhances treatment efficiency (Figure 3) [6,39]. CD44 is an overexpressed receptor on the surface of cancer cells, and it plays several roles in tumor progression [40]. Numerous targeted nanodrug delivery systems have been designed to deliver chemotherapeutic drugs to tumor sites by targeting CD44 and other receptors (Table 1).

Hyaluronic acid (HA) is widely used as a binding ligand when synthesizing CD44-targeted smart nanoparticles [60,61]. In a recent study, a polymeric nanodrug delivery system (HA/Pmet) was developed in which hyaluronic acid was employed to target lung cancer cells. The targeted nanoparticle carrier was also designed for combination therapy with cisplatin and metformin for use in lung cancer treatments. Lewis lung cancer-bearing mice were treated with targeted nanoparticles and this resulted in significantly enhanced tumor accumulation and cell proliferation inhibition, with extended overall survival when compared to free drugs without adverse side effects [44]. Wu et al. investigated another hyaluronic acid-coated polymeric nanoparticle encapsulating DTX as an anticancer drug to improve cellular uptake. This targeted nanodrug carrier showed a more than 4-fold increase in drug concentration in lung cancer cells when compared to the free drug. These effects have resulted in remarkable tumor inhibition and enhanced survival times [42].

**Figure 4:**
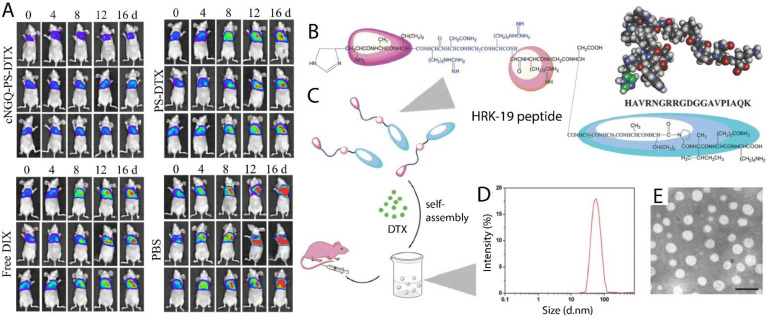
DTX containing NPs against lung cancer. (**A**) Representative bioluminescence imaging of A549 tumor-bearing mice treated with cNGQ-PS-DTX, PS-DTX, free DTX, and PBS. Adapted with permission from Ref. [63]. Copyright 2021 American Chemical Society. (**B**) Schematic and molecular structure of the HRK-19 peptide. (**C**) HRK-19 peptides and DTX mixed to form self-assembled NPs, and then intravenously injected into the tumor-bearing mouse. (**D**) Size distribution graph for DOC/peptide NPs. (**E**) Transmission electron microscope (TEM) image of DOC/peptide NPs (Dulbecco’s phosphate-buffered saline (DPBS)). The scale bar indicates 100 nm [48].

Integrins are overexpressed receptors in tumor cells. They are responsible for the crucial properties of cancer cells, from growth to metastasis [62]. To improve the DTX dose-limiting side effects, Zou et al. developed an integrin α_3_β_1_ coated artificial vesicle (cNGQ-PS) to precisely target lung cancer cells. DTX was loaded into targeted nanoparticles and examined in a lung cancer animal model. The animal study showed that cNGQ-PS-DTX had an 8-fold higher tolerability and significant drug accumulation in the cancer cells compared to free DTX, resulting in remarkable tumor suppression (Figure 4A) [63]. Another novel targeted treatment strategy to develop practical and highly specific nanodrug carriers for lung cancer cells is to employ peptides as targeting ligands on the surfaces of nanoparticles [64]. One peptide can target multiple receptors or molecules [65]. HRK-19 is a bifunctional peptide that targets integrin, CD13, and N-cadherin receptors. This peptide also contains an apoptosis-inducing motif (AVPIAQK) with antitumor properties. In an *in vitro* study, HRK-19 and AVPIAQK were used to synthesize a targeted peptide-based nanocarrier carrying DTX anticancer chemotherapeutic agents (Figure 4B–E). The synergy between DTX and AVPIAQK results in high-specificity tumor targeting, which is considerably more powerful at blocking tumor growth and long-term retention in tumors than free DTX [48].

## 3. Stimuli-Responsive Nanoparticles

### 3.1. Endogenous Stimuli

#### 3.1.1. PH-Responsive

**Figure 5:**
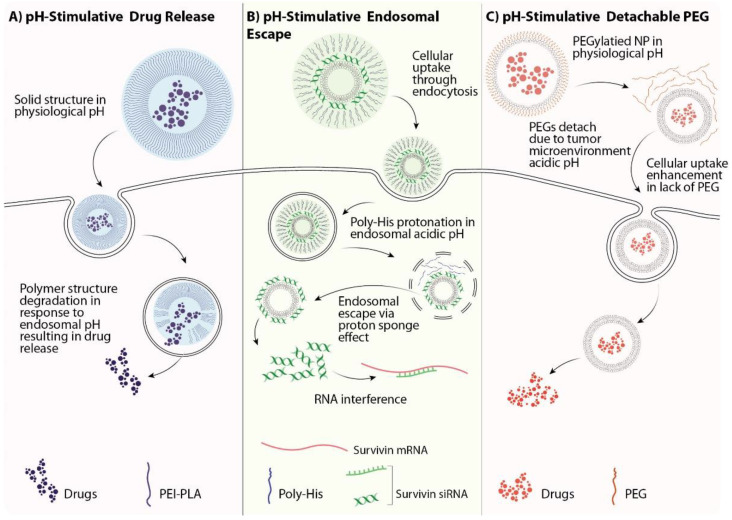
Common pH-responsive nanoparticle (NP) strategies. (**A**) The most common application of pH-responsive NPs is for controlled drug release, which is achieved using pH-simulative NPs that are stable in a physiological pH but degrade in the acidic pH of the tumor cell intracellular environment. (**B**) DNA and RNA delivery is associated with endosomal entrapment challenges. Utilizing a poly-His module in the nanoformulation can overcome this challenge via the proton sponge effect that results from poly-His protonation in endosomal acidic pH, and increases endosomal membrane permeability, resulting in endosomal escape of the NPs. (**C**) PEGylation is the most common strategy to increase the biocompatibility and circulation half-life of NPs. However, it can decrease the cellular uptake of NPs. To address this issue, a common approach is the PEGylation of nanoparticles using a pH-sensitive linker that remains stable in physiological pH and degrades in response to the acidic pH of the tumor microenvironment.

The pH of the tumor microenvironment is lower than that in healthy parts of the body due to the high glycolytic rate of cancer cells [66]. Numerous studies have taken advantage of this tumor property to develop pH-responsive nanodrug carriers to release the therapeutic agents at the tumor site to treat cancer cells precisely and effectively [67,68,69,70]. A pH-responsive nanoparticle-encapsulating DTX was developed in a study to investigate the drug release profile and anticancer activity. In the acidic pH of the tumor microenvironment, NPs significantly increased their size, resulting in faster and more efficient drug release, and consequently, they had more robust anticancer activity than the nonresponsive NPs and free DTX [71]. It has also been reported that pH-stimuli-responsive nanoparticles can release 80% of their cargo up to 15 h after administration in the acidic tumor microenvironment with no damage to the normal cells [68]. This strategy also provides greater efficiency for lung cancer, which is regularly associated with considerable adverse effects in healthy tissues [69]. Small interfering RNAs (siRNAs) are double-stranded RNA molecules that silence the expression of a specific protein through the RNA interference pathway [72]. siRNAs have emerged as anticancer therapeutics and have been widely used in recent studies [73,74]. However, these oligonucleotides must deal with endosomal entrapment. This issue can be addressed using pH-responsive NPs that are sensitive to the endosomal pH [75]. Specific polo-like kinase (PLK1) has been implicated in cell proliferation and tumor progression. It is consequently an attractive target for lung cancer treatments. PHD/LR is a hybrid polymeric nanodrug delivery system that responds to an acidic pH to deliver anti-PLK1 siRNA. This novel carrier was examined in both *in vitro* and *in vivo* human lung cancer models. The results indicated that the nanoparticles effectively released their cargo in the cells due to pH-induced rapid dissociation and there was significantly more drug action when compared with the free siRNA and loaded nanoparticles without pH-responsive properties [76]. pH-responsive NPs have also been employed for the codelivery of multiple drug treatment strategies. This nanoformulation consists of a cationic polyethyleneimine-block-polylactic acid (PEI-PLA), which is responsible for cellular uptake, and a hydrophilic copolymer (PEG-Asp) that helps to improve stability and decrease NP toxicity. When it reaches the acidic intracellular tumor microenvironment, the outer layer (PEG-Asp) detaches from the NPs to expose the cationic PEI-PLA part. Hence, NPs can escape the endosome and deliver their cargo to the cytoplasm to silence survivin expression. *In vitro* studies showed no toxicity and significant survivin mRNA suppression in the A549 cell line when compared to the non-pH-responsive NPs loaded with drugs. Moreover, the *in vivo* investigation demonstrated that the codelivery in pH-responsive NPs was an effective antitumor strategy [77]. Some common strategies to obtain the most out of pH-responsive NPs for drug delivery are illustrated in Figure 5.

#### 3.1.2. Enzyme-Responsive

**Figure 6:**
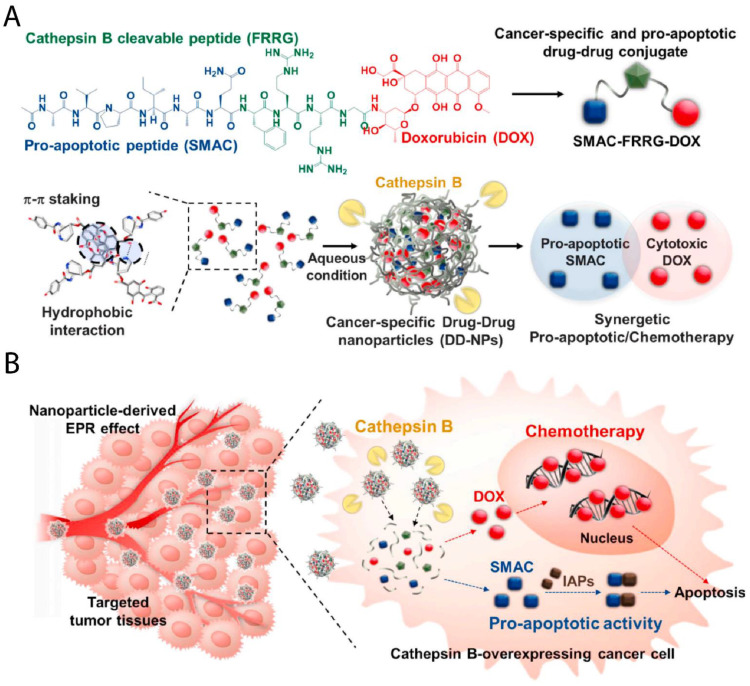
Schematic illustration of DD-NP structure and mechanism of action. (**A**) Molecular and schematic structure of DD-NPs. (**B**) DD-NPs accumulate in tumor tissue through the enhanced permeability and retention effect (EPR). Cathepsin B then cleaves the drug–drug interactions to release DOX and SMAC into the cancer cells. Reproduced with permission from Ref. [84].

Numerous types of nanomaterials (e.g., polymers and liposomes) have been designed to decompose and release their cargo in the presence of a specific overexpressed enzyme that is found in tumor sites [78]. For example, esterase is an overexpressed enzyme in the tumor microenvironment, and several esterase-responsive NPs have been developed and examined for the treatment of cancer [79]. Tam et al. developed o(L + DLA)10-GEM micelle NPs and evaluated their antitumor efficacy using an A549 human lung cancer xenograft model. The micelle NPs contain prodrugs of gemcitabine and release their cargo in the presence of esterase enzymes at tumor sites. This study demonstrated that the efficacy of o(L + DLA)10-GEM micelles was greater than that of gemcitabine and saline alone. Moreover, the NPs showed higher stability and sustained drug release [80]. Matrix metalloproteinases (MMPs) are overexpressed enzymes that play an essential role in the growth and metastasis of cancer cells [81]. GNP-DTX/Qu/IMA is a gelatin-modified cationic nanostructured lipid carrier (NLC) system produced for the codelivery of three anticancer drugs (DTX, quercetin, and imatinib) into 4T1 tumor cells. Once the nanostructure reaches the tumor area, the gelatin layer of the NPs is degraded by MMP9, leading to the release of imatinib molecules into the gelatin layer and the tumor ECM, respectively. The cationic NLC cores containing two other drugs then enter the cells. After a single administration, this nanoplatform decreased tumor interstitial fluid pressure (IFP) and prevented cancer growth and metastasis in 4T1 tumor-bearing mice [82]. Cathepsin B is another target for enzyme-responsive NPs and has many roles in cancer progression, such as cell growth, cell migration, and angiogenesis [83]. Shim et al. synthesized drug–drug enzyme-responsive nanoparticles (DD-NPs) by conjugating a cathepsin B-cleavable peptide with DOX and SMAC peptide, which facilitates intracellular delivery and has anticancer activity. In cancer cells, overexpressed cathepsin B clefted the nanoformulation and released DOX and SMAC (Figure 6). The synergy of DOX and SMAC reduced cancer growth and metastasis with minimal side effects when compared with traditional chemotherapy [84].

#### 3.1.3. ROS-Responsive

Abnormal concentrations of reactive oxygen species (ROS) is a recognized cancer initiation factor. The ROS concentrations in the tumor microenvironment have been shown to be significantly higher than those in normal cells [85,86]. Consequently, many NPs have been designed using this difference in ROS as a trigger for drug release [87,88]. Curcumin is an anticancer compound, but its anticancer properties are limited by its low solubility in water and instability in the physiological environment. To overcome these issues, curcumin-coordinated ROS-responsive (PHHC) NPs have been utilized to deliver curcumin [89]. In this study, the nanoformulation released curcumin in response to the tumor intracellular ROS after cellular uptake, resulting in remarkable lung cancer cell apoptosis. An *in vitro* study indicated that PHHC NPs can effectively induce apoptosis in tumor cells in response to ROS, and they showed minimum cytotoxicity in the presence of ROS inhibitors, indicating that PHHC NPs affected tumor cells with high ROS levels, but not normal cells [90].

#### 3.1.4. Redox-Responsive

**Figure 7:**
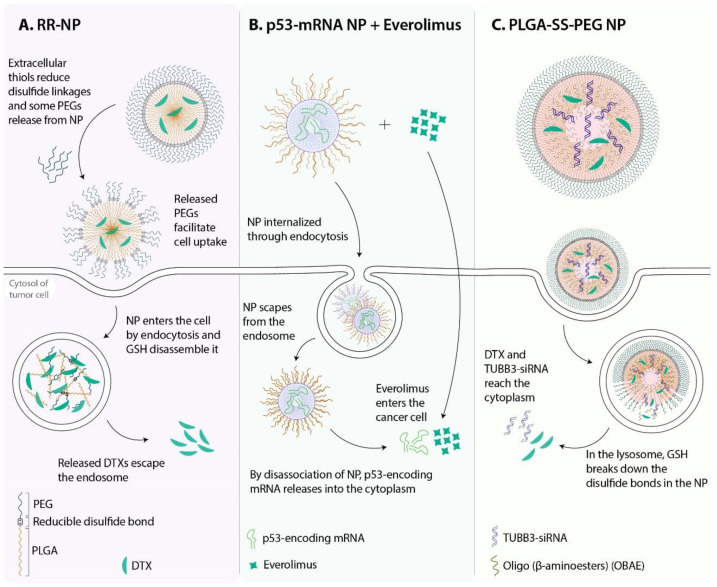
Redox-responsive NPs. Various nanoplatforms have been developed to deliver anticancer agents into cancer cells. (**A**) RR-NP is a polymeric NP designed to carry DTX selectively into tumor cells. (**B**) Redox-responsive NPs can be used to deliver nucleic acid anticancer agents to tumor tissues. The p53-mRNA NP is a nanocarrier that delivers p53-encoding synthetic messenger RNA (mRNA). P53-null tumor cells that receive p53-encoding genes become sensitive to everolimus (a mammalian target of rapamycin (mTOR) inhibitor). A combination of p53-mRNA NP and everolimus showed efficient antitumor function against NSCLC. (**C**) PLGA-SS-PEG NP is a spherical nanoformulation with a size of approximately 150 nm. This redox-responsive NP is developed for codelivery of DTX and TUBB3 siRNA. The NP enters the cell by endocytosis, and in the lysosome, GSH induces breakage of the disulfide bonds and disassembly of the NP. Consequently, DTX and TUBB3 siRNA escape from the lysosome and perform their anticancer activity in the cytosol.

**Figure 8:**
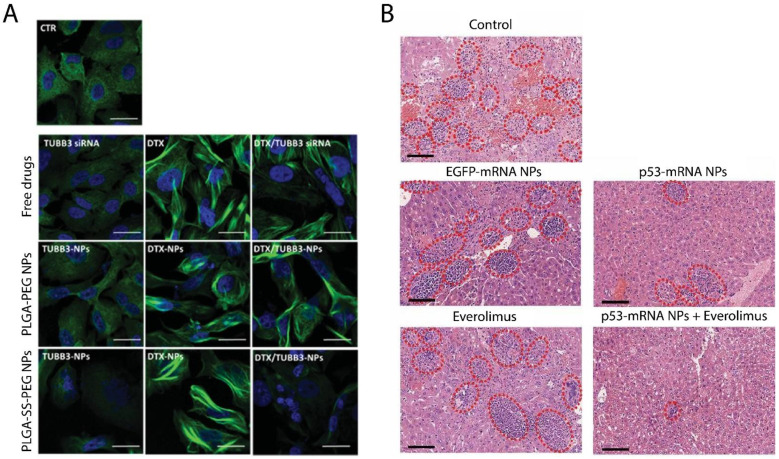
Redox-responsive NPs in combination with anticancer agents. (**A**) Immunofluorescence microscopy images of A549 tumor cells treated with PLGA-SS-PLGA, PLGA-PEG NPs, and free drugs (βIII-tubulin was stained in green and nuclei were stained with Hoechst 33,258 in blue). The scale bars indicate 10 μm [100]. (**B**) Metastatic clusters (showed in red dotted ovals) in liver tissue after treatment with p53-mRNA NPs + everolimus, p53-mRNA NPs, everolimus, and EGFP-mRNA NPs. The scale bars represents 100 μm [98].

There are significant differences between the redox potentials found in tumors and those of healthy tissues and between intracellular and extracellular fluids. For instance, the concentration of reductive glutathione (GSH) tripeptides in tumor cells is 100–1000 times higher than that in blood. This difference can be used to help target drug delivery to cancer cells [26]. Several redox-triggered NPs have been reported in combination with chemotherapeutic drugs [91,92,93,94]. In a recent *in vitro* study, redox-sensitive poly(lactic-co-glycolic acid) (PLGA)-PEG NPs were used to deliver DTX into lung cancer cells via the pulmonary route. The 2D and 3D cell culture models showed that redox-responsive NPs were more effective than non-redox-responsive NPs (Figure 7A) [95]. Lyer et al. developed cisplatin-loaded GSH-sensitive NPs (CGPU) to effectively target lung cancer. GSH-cleavable disulfide bonds led to a GSH dose-dependent release of cisplatin from NPs for up to 5 days. CGPU was cyto-compatible with healthy lung cells and was more effective in preventing tumor growth in mouse models than free drugs [96]. PTX and berberine conjugated with disulfide bonds and formed NPs with GSH-responsive and mitochondria-targeting behavior, providing promising results for the codelivery of anticancer drugs in redox-responsive NPs [97]. Redox-responsive NPs have also been used to deliver different types of RNAs. For example, Kong et al. reported a redox-responsive lipid-polymer hybrid NP that could codeliver p53 mRNA and everolimus (Figure 7B). Employing this nanodrug delivery system led to the restoration of p53 in p53-null Hep3B HCC and H1299 NSCLC. The restoration of p53 results in sensitization to everolimus in these cells and applying both agents was more effective than using p53 mRNA alone. Finally, p53 mRNA hybrid NPs prevented tumor cell growth by inducing cell apoptosis and suppressing the cell cycle in the G1-phase (Figure 8A) [98]. Class III β-tubulin is a cytoskeletal protein associated with taxane resistance [99]. Silencing the βIII-tubulin encoder gene, TUBB3, is considered to be a potential approach to sensitize tumor cells to taxanes. DTX/TUBB3-siRNA is a redox-responsive nanocarrier produced for the codelivery of DTX and TUBB3 siRNA (Figure 7C). Redox-triggered NPs induced more TUBB3 gene silencing and higher cell uptake when compared to non-redox-responsive NPs (Figure 8B). Further examinations demonstrated that DTX/TUBB3-siRNA NPs are suitable for delivery through the pulmonary route [100].

### 3.2. Exogenous Stimuli

#### 3.2.1. Photoresponsive

Interest in photoresponsive nanodrug delivery systems has increased as they are noninvasive and have controllable temporal and spatial drug release characteristics [101]. This strategy offers numerous advantages for the reduction of adverse drug reactions and to improve controlled biodistribution [24]. Promising outcomes in photoresponsive nanoformulations investigated in preclinical and clinical studies led to the first FDA-approved light-sensitive liposome NPs, known as Visudyne^®^, in 2000 to treat disorders associated with the eye [102]. Photoresponsive nanotherapeutics act through two main mechanisms: photodynamic therapy (PDT), a local ROS generation in response to radiation with specific wavelengths that can lead to cell apoptosis or necrosis, and photothermal effect (PTT), light-triggered hyperthermia that can lead to liposomes or lipid-based micelles for facilitated drug release, increasing chemotherapeutics cytotoxicity, and also driving the cell to necrosis and apoptosis in higher temperatures [103].

Chlorin e6 (Ce6) is a well-known photosensitizer in photodynamic therapy (PDT) that has a high efficacy and low toxicity in its inactive form. In the presence of light, Ce6 produces ROS, leading to the elimination of cancer cells. In a study, Kumari et al. conjugated Ce6 with a polymeric nanoparticle (mPEG-PLA-Ce6) to increase cellular uptake. The phototoxicity of mPEG-PLA-Ce6 against A549 cells was higher than that of the free drug, and NPs showed higher antiproliferative effects, greater cellular uptake, and prominent cell apoptosis [104].

A number of studies have investigated the use of photosensitive NPs combined with chemotherapeutic agents to achieve both anticancer activities and the controlled release of photosensitizers [105]. FePt-Cys is a photoresponsive nanoparticle designed to have high water solubility, good biocompatibility, uniform distribution, and dispersion. An *in* *vitro* study indicated that NPs increased ROS levels and caused apoptosis in tumor cells. Furthermore, FePt-Cys NPs reduced tumor invasion and migration by decreasing MMPs. In addition, the effectiveness of FePt-Cys NPs in combination with cisplatin was measured, and the NPs showed greater efficiency when in synergy with cisplatin and radiation in disturbing angiogenesis and tumor growth in tumor-bearing mice models [106]. In another study, a gemcitabine reduced graphene oxide NP, termed as GEM-rGO, was developed as a chemo photothermal agent. The highest antitumor performance was achieved in human lung carcinoma when near-infrared radiation (NIR) was applied to GEM-rGO NPs [107].

Photoresponsive NPs in combination with therapeutic agents have shown promising results in preventing the lung metastasis of breast cancer [94,108,109]. Luo et al. used a porphyrin−phospholipid (PoP) as a photoresponsive agent and ^64^Cu as a radiolabelling factor in DOX-loaded liposomes. The NPs released DOX in the presence of 665 nm light emissions, effectively preventing the primary growth of 4T1 cell lines. In addition, other investigations have demonstrated that the accumulation of DOX in the lungs of tumor-bearing mice was higher than that in healthy mice. It is suggested that the expansion of DOX in the lungs is due to the EPR effects. Further adjustment of the NP size and surface characteristics could reduce its accumulation in nonmetastatic lungs [110].

#### 3.2.2. Ultrasound-Responsive

**Figure 9:**
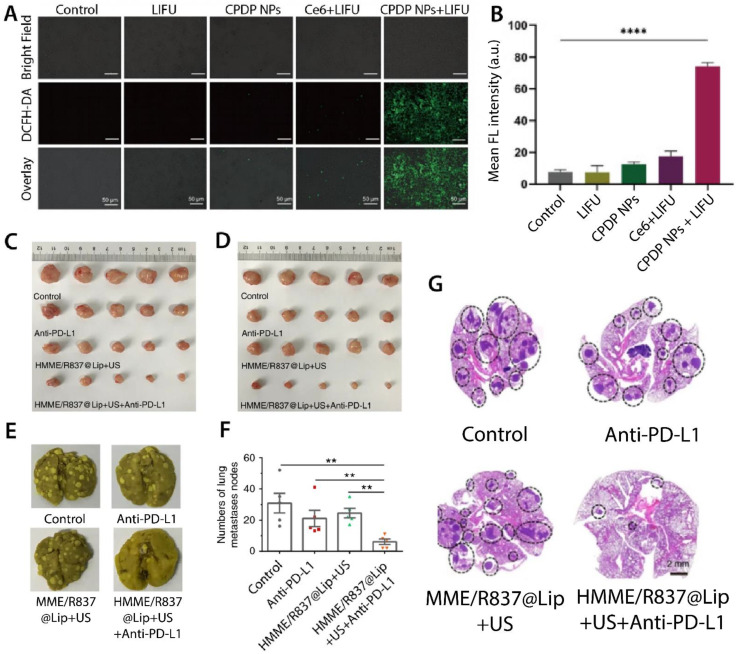
The efficiency of different ultrasound-responsive NPs against lung cancer. (**A**) Confocal laser scanning microscopy images of ROS generation in CPDP NPs + LIFU, Ce6 + LIFU, CPDP NPs, and LIFU treatments (scale bars; 50 μm); and (**B**) FL intensity analysis of these groups (**** *p* < 0.0001, n = 3) [112]. (**C**) Photograph of primary tumors treated with HMME/R837@Lip + US + Anti-PD-L1, HMME/R837@Lip + US, and Anti-PD-L1 and (**D**) distant tumors in mice treated with these NPs. (**E**) Photograph of metastatic nodules in mice in different groups and (**F**) the number of metastatic tumors in the lung counted under the microscope (** *p* < 0.01). (**G**) Histological examination of lung metastasis in different groups (hematoxylin and eosin) [114].

Ultrasound is a safe and noninvasive approach that plays a crucial role in both diagnostic and therapeutic applications. To achieve the selective elimination of tumors, sonodynamic therapy (SDT) has recently been investigated. It has beneficial features such as deep tumor penetration, nonionizing properties, and low costs, making it an ideal approach for designing stimuli-responsive NPs. Employing gold or magnetic NPs with ultrasound showed selective performance against cancer cells [111]. Ultrasound-sensitive NPs have been used as drug delivery systems for various anticancer agents. Zhang et al. encapsulated DOX, Ce6, and perfuoropentane in a PLGA core-shell structure (CPDP NPs) and examined its anticancer activity against the 4T1 cell line. CPDP NPs in the presence of low-intensity focused ultrasound (LIFU) irradiation indicated a potent antimetastatic ability by which lung metastatic nodules were considerably decreased [112] (Figure 9A,B).

Another study developed ultrasound-sensitive chitosan-deoxycholic acid NPs to deliver siRNA into A549 lung cancer cells. The results demonstrated that there was a positive correlation between the energy of ultrasound irradiation and treatment efficacy. When compared to the control group, a 4-fold decrease in cell viability was observed using this nanoformulation, demonstrating the effectiveness of the method for drug delivery and gene silencing [113].

HMME/R837@Lip is a therapeutic NP designed to combine SDT with immunotherapy. Hematoporphyrin monomethyl ether (HMME) was used to produce ROS in the presence of ultrasound irradiation. Imiquimod (R837) has also been used as an immune adjuvant to provoke an immune response after applying SDT. HMME/R837@Lip-augmented SDT combined with anti-PD-L1 blockade inhibited 4T1 tumor growth by 95%, decreased distant tumor growth by 83%, and significantly prevented lung cancer metastasis in tumor-bearing mice. The effects of HMME/R837@Lip + SDT + anti-PD-L1 with the three control groups are shown in Figure 9 [114].

### 3.3. Multiresponsive Nanodrug Delivery Systems

Multiple combination strategies for smart nanodrug delivery have attracted much attention owing to their numerous advantages, such as improved specific drug delivery capability, increased control over the drug release profile, and synergistic effects. These multiple-responsive nanodrug delivery systems are generated with various types of nanomaterials and are sensitive simultaneously to different physical, chemical, and biological stimuli, providing the capacity for precision medicine [115,116].

In a previous study, PPT/D(DMA)@DOX, a pH and ROS pH-responsive polymeric micelle nanodrug delivery system loaded with DOX, was produced and compared with PPT/D(SA)@dox. The low pH of the tumor microenvironment caused changes in the nanoparticle surface charge from negative to positive, which triggered and promoted nanoparticle cellular uptake. Moreover, the ROS concentration inside the cell triggers TOS, an analogue of vitamin E co-encapsulated in the NPs, enhancing the ROS to activate complete drug release. It has been shown that PPT/D(DMA)@DOX has better antitumor activity both *in vitro* and *in vivo*. This novel DOX-loaded pH/ROS-responsive nanocarrier improved the biodistribution profile and tumor accumulation when compared to PPT/D(SA)@DOX [117]. Another study synthesized GSH and H_2_O_2_ responsive polymeric-base nanoparticles with a diselenium bond (Se-Se) in the main chain to improve the encapsulation efficiency of cisplatin. After cellular uptake, the Se-Se bonds reacted with GSH and H_2_O_2_, resulting in nanoparticle degradation and drug release. The therapeutic impacts of this nanoformulation were evaluated in different types of cancers, including lung cancer. *In vitro* and *in vivo* studies have shown that NPs can effectively induce apoptosis, reduce tumor size, and overcome multidrug resistance which are the critical failures of lung cancer chemotherapy [118].

Recently, pH and enzyme dual-responsive NPs have been reported. PM@THL is a pH and enzyme-responsive nanoparticle designed to target breast cancer with lung metastasis. PM@THL is a drug delivery system responsible for the integration of a three-in-one system: (i) HY19991 (a PD-1/PD-L1 interaction inhibitor), (ii) thioridazine (an anticancer stem cell agent), and (iii) PTX. HY19991 and thioridazine release was triggered by MMP-9 in the tumor microenvironment, and the presence of both an acidic pH and MMP-9 results in PTX release. This study suggests that PM@THL NPs can reduce metastatic lung nodules by 97.64% and induce T cell infiltration into tumors [119].

PAHD is a pH and enzyme dual-responsive NP that has been evaluated against breast cancer and lung metastasis. This nanocomplex codelivers DTX and aspirin prodrugs into a cationic polyethyleneimine (PEI)-PEG copolymer shell. Aspirin is a nonsteroidal drug with antimetastatic properties against different types of cancer [120]. The PEG-PEI molecules separate from the NPs because of the tumor acidic microenvironment. Thus, free cationic PEG-PEI facilitates NP cellular uptake. Then, heparanase (an overexpressed enzyme in cancer cells) degrades DTX prodrug to release aspirin and DTX. PAHD caused the infiltration of CD8+ T cells into tumor sites and inhibited the metastasis of 4T1 cells by 98.4% [121].

The development of nanoparticles that are responsive to more than two stimuli has recently been reported. PEG-M-PPMT NPs are pH-, enzyme-, and ROS-responsive NPs that carry sorafenib (an anticancer drug) and chlorine e6, which is a photosensitizer. These NPs lose their PEG coronas in the presence of MMP-2 in the tumor microenvironment, resulting in shrinkage that facilitates NP cellular uptake. Then, the NP degrades in the endosomal acidic pH and tumor cell ROS concentration, leading to sorafenib and chlorine release. Ce6 generates greater ROS by activation through external laser irradiation. As a result of elevated cellular ROS concentrations, tumor cells are driven to apoptosis. In addition, increasing the ROS concentration promotes drug release. *In vivo* and ex vivo evaluations of PEG-M-PPMT NPs indicate that these NPs can completely eradicate approximately 29% of all tumors without toxicity to healthy organs, including the heart, liver, lung, spleen, and kidney [122]. An overview of some of the recent studies using dual and multiresponsive NPs for lung cancer treatment is shown in Table 2.

## 4. Targeted Stimuli-Responsive NPs

In recent years, designing NPs with both targeting agents and stimuli-responsive properties is a novel method that has been suggested. NPs modified to be simultaneously targeted and stimuli-responsive demonstrate better cellular uptake and antitumor efficacy than NPs modified for only a single trait.

In a recent study, gold nanostars were coated with bovine serum albumin to increase NP stability and encapsulated IR 780 iodide. The polypepeptide (Ac-GPLGIAGQ) MPP substrate was used to target overexpressed MMP2 in lung cancer cells. IR 780 iodide is an FDA-approved NIR fluorescence dye with PDT and photothermal therapy (PTT) properties in response to laser irradiation, causing ROS generation and local heating, respectively, both of which can lead to tumor cell death. Moreover, the combination of gold nanostars and IR 780 iodide resulted in an NIR/photoacoustic imaging tool. *In vitro* and *in vivo* results showed that GNS@BSA/I-MMP2 NPs have much better cellular uptake, antitumor (93% tumor volume decrease) efficacy, and only minor organ damage when compared to the free IR 780 iodide and nontargeted IR 780 encapsulated NPs [131].

**Figure 10:**
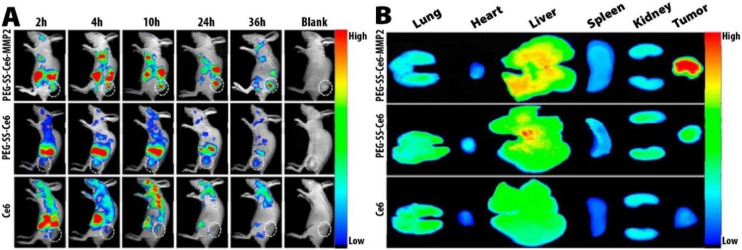
In vivo biodistribution and tumor accumulation of PEG-SS-Ce6-MMP2 NP compared with free Ce6 and PEG-SS-Ce6 NPs. (**A**) *In vivo* whole-body fluorescence imaging after intravenous injection of free Ce6, PEG-SS-Ce6, and PEG-SS-Ce6-MMP2 NPs to A549 tumor-bearing mice in a time dependent manner. (**B**) *In vitro* fluorescence images of drug accumulation in major organs and tumors of mice, 36 h after the intravenous injection of free Ce6, PEG-SS-Ce6 NPs, or PEG-SS-Ce6-MMP2 NPs. Adapted with permission from Ref. [132]. Copyright 2021 American Chemical Society.

PEG-SS-Ce6-MMP2 is another NP targeted to MMP2 and responsive to cellular GSH and laser irradiation that was developed for PDT and NIR imaging simultaneously. The structure of this smart nanocarrier was based on Ce6 conjugation to an MMP2-cleavable polypeptide and coated with cleavable PEG via redox-sensitive disulfide bonds. After cellular uptake through the MMP2-mediated targeting mechanism, the high cellular GSH level causes Ce6 release from the NPs. In response to laser irradiation, Ce6 generates ROS which results in cell apoptosis. Cellular uptake assays using confocal laser scanning microscopy (CLSM) have shown that PEG-SS-Ce6-MMP2 had more potent Ce6 red fluorescence, hence better cellular uptake than PEG-SS-Ce6 (nontargeted PEGylated Ce6) and free Ce6. In addition, PEG-SS-Ce6-MMP2 showed a significant PDT effect after laser irradiation in an *in vitro* lung cancer model. In the *in vivo* study, the nanoparticles demonstrated excellent tumor accumulation with minor organ damage and completely suppressed the tumors without recurrence within 30 days in tumor-bearing mice (Figure 10) [132].

**Figure 11:**
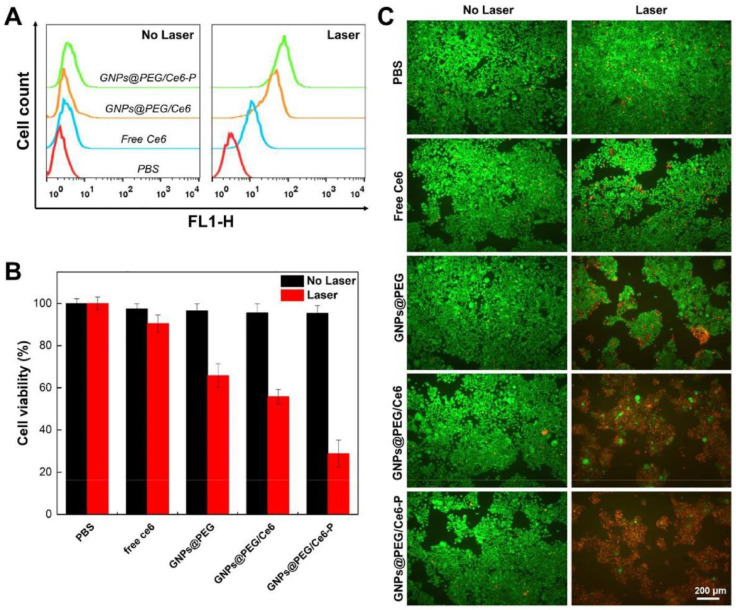
Impact of PBS, free Ce6, nontargeted NP, and targeted NP on HCC872 cell viability. (**A**) ROS generation of HCC827 cells after treatment with PBS, free Ce6, nontargeted NP, and targeted NP for 12 h in the absence and presence of laser irradiation. (**B**) Different NP effects on the viability of HCC827 cells, with and without laser irradiation. (**C**) Dead/live assay on HCC827 cells with different NPs before and after laser irradiation (green dots and red dots suggest live and dead cells, respectively). Reproduced with permission from Ref. [134].

Another study combined the effect of MMP2 targeted photosensitive Ce6 with pH-responsive cis-aconitic anhydride-modified DOX (CAD). In this study, gold nanoclusters were decorated with PEG to increase their stability and biocompatibility, they were also coated with MMP2 polypeptides to specifically target the tumor tissue. The nanoparticles were loaded with Ce6 and CAD to combine the matrix and chemotherapy. Cellular uptake evaluation using CLSM indicated that the smart nanodrug delivery system, namely CDGM, enhanced both DOX and Ce6 cellular uptake when compared with the nontargeted nanoparticles and combined free DOX and Ce6. The biodistribution and tumor inhibition assessments of tumor-bearing mice demonstrated that CDGM NPs have excellent tumor accumulation and successfully inhibit tumor growth. No significant body weight loss or organ damage was observed in the 21-day period after injection, indicating that CDGM NPs are relatively safe [133]. These promising results led to another study in which PD-L1 peptides were employed to synthesize targeted stimuli-responsive PEGylated-gold nanoparticles encapsulating Ce6 for PDT and real-time imaging. The GNPs@PEG/Ce6-P showed better cellular uptake and PDT effects in response to laser irradiation when compared to free Ce6 and nontargeted NPs (Figure 11). *In vivo* evaluation showed effective drug accumulation in the tumor and inhibition of tumor growth with minimal organ damage and body weight loss [134].

**Figure 12:**
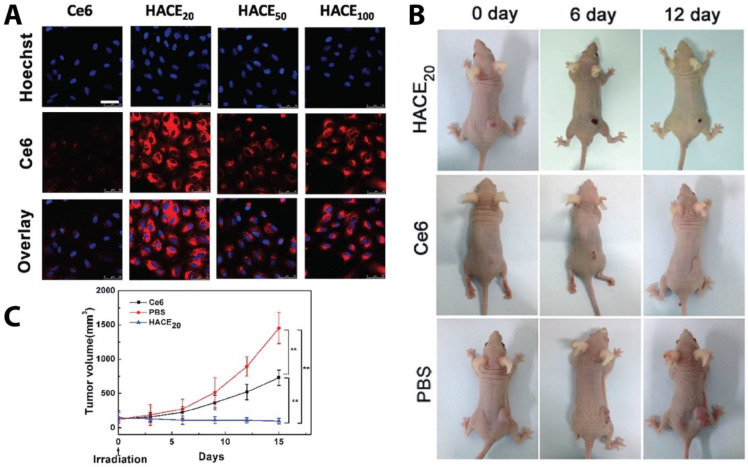
HACE NP and free Ce6 cellular uptake and tumor suppression. (**A**) Confocal microscopy images of free Ce6 and HACE NPs (different size and mass) cellular uptake by A549 cells. Scale bar, 50 μm. (**B**) Tumor images and (**C**) tumor volumes for A549 tumor-bearing mice after 15 days of treatment with PBS, free Ce6, and HACE_20_ (** denotes *p* < 0.01). Reproduced with permission from Ref. [135].

A recent study reported a smart hyaluronidase-activated theranostic micelle (HACE) based on HA attached to Ce6, a photosensitizer, that targeted the CD44 HA receptor and was sensitive to laser irradiation and the hyaluronidase enzyme. After CD44 receptor-intermediate endocytosis, hyaluronidase hydrolyzes the nanoparticles’ HA backbone and releases Ce6 into the cytoplasm, for PDT in response to laser irradiation. The *in vitro* investigation showed that the nanoparticles had a greater cellular uptake and PDT effect than the free Ce6. *In vivo* studies also indicated that there was increased nanoparticle accumulation in the tumor when compared to the free Ce6. The nanoformulation effectively inhibited tumor growth and decreased tumor volume within 15 days (Figure 12) [135].

DTX/PPN@PPY@HA is another NP that takes advantage of HA as a targeting agent. This NP was based on a fatty acid phase-changing core, photoresponsive polypyrrole outer layer, HA, and DTX combination. In tumor cells, polypyrrole stimulation with laser irradiation causes local hyperthermia, resulting in fatty acid core melting and heat-triggered drug release. This study suggested that NP DTX/PPN@PPY@HA had excellent cellular uptake and remarkable photothermal chemotherapeutic effects, while the blank PPN@PPY@HA showed no cytotoxicity *in vitro* and no damage to normal tissues in animal studies. In addition, *in vivo* assessments demonstrated that the intratumoral injection of NPs can completely eradicate the tumor, and its intravenous injection can inhibit tumor growth and decrease tumor mass [136]. The preclinical studies on targeted stimuli-responsive NPs are summarized in Table 3.

## 5. Clinical Trials

Numerous clinical studies have been conducted to evaluate the efficacy and safety of nanodrug delivery systems, however, to date, the FDA has approved only a few for use with lung cancer patients. Genexol-PM, a polymeric micelle formulation of PTX, and nab-PTX are two nanotechnology-based drugs currently used to treat lung cancer [6]. The majority of nanomedicines that are used in clinical trials are targeted nanocarriers. Targeted nanodrug delivery strategies have been shown to offer practical pharmacokinetics, maximum tumor accumulation with minimum adverse effects, and lower required doses when compared to free anticancer drugs [161]. A summary of some of the clinical studies utilizing nanotherapeutics for the treatment of lung cancer is presented in Table 4.

BIND-014 is a polymeric nanocarrier carrying DTX in hydrophilic polyethylene glycol corona decorated with ligands that target the prostate-specific membrane antigen (PSMA) [162]. Overexpression of PSMA is not limited to prostate cancer and it has been identified in other types of cancer, especially NSCLC [163]. In preclinical studies, BIND-014 was more efficient than solvent-based DTX and nontargeted DTX-loaded NPs in reducing the mean tumor weight and tumor suppression. Recently, two clinical studies, both in the phase II stage, investigated the impact of BIND-014 on patients with lung cancer. In the first study (NCT02283320), BIND-014 was evaluated in patients with KRAS-positive or squamous cell NSCLC, and the other (NCT01792479) examined the efficacy and safety of this nanotherapeutic in patients with advanced NSCLC [164].

TargomiRs are nonliving bacterial minicells loaded with an miR-16-based mimic microRNA (miRNA). Panitumumab, a human monoclonal antibody, selectively binds to overexpressed EGF receptors to efficiently target tumor cells [165,166]. There is only one available phase I trial investigation of TargomiRs, which evaluates the application of these NPs as a second or third line treatment for patients with recurrent malignant pleural mesothelioma and NSCLC (NCT02369198).

SGT-53 is another active targeted NP that delivers the tumor suppressor gene TP53 to tumor cells and restores the function of the p53 protein [167]. p53 is a tumor suppressor protein that is altered in most types of cancer. In this nanocomplex, a single-chain antibody fragment was used to target the transferrin receptor. Phase I and phase IB trials have shown favorable safety profiles [168]. There is a new early phase I trial being conducted to evaluate SGT-53 NPs in pediatric patients with recurrent or progressive CNS malignancies (NCT03554707). A recent study showed that using a combination of anti-PD1 immunotherapy with SGT-53 NPs is a practical approach against NSCLC in mouse models [169]. According to this study, SGT-53 could be potent against lung cancer and should be investigated further in future trials.

## 6. Future Perspective

Smart nanotechnology-based drug delivery systems provide new hope that we may be able to overcome the current limitations of the anticancer agents used for lung cancer treatment. As we see in the case of Abraxane, the advantages of nanotechnology-based drug delivery were so significant that it ended the monopoly of a traditional chemotherapeutic agent (Taxol). Even though Abraxane gained considerable success, it solely uses passive targeting strategy to reach the tumor site, which, compared to new “smart” NPs, has fewer advantages. Furthermore, this novel strategy could play a key role in precision and personalized medicine in the near future. However, there are still many challenges that need to be addressed in the development of nanotherapeutics with multifunctionalities to improve cancer nanomedicine clinical trials. As NPs become more complicated in terms of structure and formulation, industrial scale-up production becomes more challenging. In addition, it is more difficult to control the physicochemical properties of these smart nanoformulations due to their complexity. Discovering the exact mechanism of smart NPs in the human body seems crucial for the further growth of this field. Differences in endogenous factors (e.g., pH and enzymes) between individuals is another challenge since it could alter the efficiency of smart NPs. This issue became problematic in the case of stimuli-responsive NPs that target one or more endogenous stimuli. In contrast, controlling exogenous stimuli are more straightforward, but optimization is needed to reduce damaging normal tissue and adjust tissue penetration depth. Comprehensive standard protocols for the toxicological profiling of nanoformulations could help to determine the critical physical or chemical properties of NPs which cause their toxicity. Novel nanoformulations and safer strategies for application could result from optimizing the physicochemical properties of NPs while also increasing their therapeutic efficacies. New approaches, such as machine learning and in silico methods, could be employed as a powerful tool in nanomedicine to develop novel nanoformulations and find suitable treatment strategies.

## Figures and Tables

**Table 1:** Recently developed targeted nanotherapeutics for lung cancer.

Nanotherapeutic Name/Abbreviation	Target Cell	Targeting Agent	Target	Therapeutic Agent(s)	Ref.
Docetaxel (DTX)-loaded hyaluronic acid (HA) nanocapsules	A549	HA	CD44 receptor	DTX	[41]
DTX-HPLGA	A549	HA	CD44 receptor	DTX	[42]
HA@DOX@CNC	A549	HA	CD44 receptor	Doxorubicin (DOX)	[43]
HA-CDDP/PMet	4T1—Lewis lung carcinoma LLC—HepG2	HA	CD44 receptor	Cisplatin—Metformin	[44]
Apt-Co-NPs	A549—SK-MES-1	HA	CD44 receptor	DOX—Cisplatin	[45]
DTX-loaded ImI-PMs	A549	α-Conotoxin ImI	α7-nAChR	DTX	[46]
CHC/DMC-CDDP/anti-CD133	A549-ON	CD133-antibody	CD133 receptor	Cisplatin—demethoxycurcumin	[47]
DOC/peptide	A549	HRK-19 peptide	Integrin α_v_β_3_—CD13—E-cadherin	DTX	[48]
cRGD-PLGA@DOX	A549—HeLa	Cyclic arginine-glycine-aspartic acid polypeptide (cRGD)	Integrin α_v_β_3_	DOX	[49]
cRGD-LPP-Dox	A549	cRGD	α_v_β_3_ Integrin	DOX	[50]
RGDfC-Se@DOX	A549	Cyclic peptide RGDfC	α_v_β_3_ integrin	DOX	[51]
EGF DTX/RSV LPNs	HCC827—NCIH2135—HUVEC	Epidermal growth factor (EGF)	EGF receptor	DTX—resveratrol	[52]
DTX-CS-TPGS-CTX-NPs	A549	Cetuximab	EGF receptor	Cetuximab—DTX	[53]
LPs-DTX-FA	A549—SPCA1	Folic acid	Folate receptor	DTX	[54]
^64^Cu-NOTA-GO-FSHR-mAb	cbgLuc-MDA-MB-231	Monoclonal antibody (mAb)	Follicle-stimulating hormone receptor (FSHR)	DOX—mAb	[55]
C-DVM	4T1	CREKA peptides	Fibronectin	DOX—Vinorelbine	[56]
DOX-DNA	A549—4T1	AS1411 aptamer	Nucleolin	FOXM1 aptamer—DOX	[57]
DL-NCs	A549/CDDP	D-α-tocopheryl polyethylene glycol succinate (TPGS)—transferrin	Transferrin receptor	Cisplatin—TPGS	[58]
NAG-Dend-CPT	A549	N-acetylD-glucosamine (NAG)	Glucose transporters—lectin receptors	Camptothecin	[59]

**Table 2:** Recent smart nanotherapeutics developed for lung cancer that are responsive to two or more stimuli.

Nanotherapeutic Name/Abbreviation	Stimuli	Anticancer Agent(s)	In Vitro	In Vivo	Ref.
PAHD	pH—enzyme (Heparanase)	DTX—aspirin	4T1	4T1 tumor-bearing mice	[121]
PM@THL	pH—enzyme (matrix metalloproteinase (MMP))	PTX- thioridazine—HY19991	MCF-7	Mice bearing metastatic MCF-7 tumors	[119]
Fe3O4@SiO_2_@DOX	pH—Photothermal	DOX	A549	-	[123]
mPEG-PAAV/IR780 + DOX micelle	pH—Photothermal	DOX	4T1	4T1 tumor-bearing mice	[124]
TOCM	pH—redox	Cisplatin—TPGS	A549DDP	H22 tumor-bearing mice	[125]
PTXL-ss-PMAGP-GEM/NAG NLC	pH—redox	Gemcitabine—Paclitaxel	A549—LTEP-a-2	A549 tumor-bearing nude mice	[126]
PSPm(PTX/CDDP)	pH—redox	PTX—Cisplatin	NCI-H520—CRL-5802—NCI-H358	-	[127]
DOX-N-NPs	pH—redox	DOX	A549—MCF-7	MCF-7 tumor-bearing mice	[128]
GP-NA	pH—redox	Ultra-small platinum nanoparticles—Gem	A549—NCI-H1299	NCI-H1299 tumor-bearing mice—A549 tumor-bearing nude mice	[129]
PPT/D(DMA)@DOX	pH—ROS	DOX	A549	A549 cell tumor-bearing mice model	[117]
SFR/Ce6-loaded PEG-PPMT	pH—ROS—enzyme	Sorafenib chlorin e6 (Ce6)	A549	A549 xenografted in nude mice	[122]
ZnPc/CPT-TPPNP	ROS—photodynamic	Camptothecin	NCI-H460	NCI-H460 tumor-bearing mice	[130]
NP(Se)	Redox—H2O2	Cisplatin prodrug	A549, A549DDP, 7404DDP	Patient-derived xenograft models of hepatic carcinoma and multidrug-resistant lung cancer	[118]

**Table 3:** Recent smart nanotherapeutics for lung cancer that are simultaneously targeted and stimuli-responsive.

Nanotherapeutic Name/Abbreviation	Targeting Agent	Target	Stimuli	Drug	Therapy	Application	Cell/Animal Model	Ref.
HA-ZnO-DOX	Hyaluronic acid	CD44 receptor	pH	DOX	Chemotherapy	Therapy	A549 cell line	[137]
HACE	Hyaluronic acid	CD44 receptor	Photo	Ce6	Photodynamic therapy	Therapy and imaging	A549 cell line, A549 tumor-bearing nude mice model	[135]
DOX/HA-ss-DOX micelles	Hyaluronic acid	CD44 receptor	pH, redox	DOX	Chemotherapy	Therapy	A549 cell line, xenograft model	[138]
DTX/PPN@PPY@HA	Hyaluronic acid	CD44 receptor	Photo, thermo	DTX—Polypyrrole	Chemotherapy, photothermal therapy	Therapy and imaging	A549 cell line, 4T1 tumor-bearing mice model	[136]
HPGBCA	Hyaluronic acid	CD44 receptor	Photo, enzyme, ROS	Afatinib, Ce6	Chemotherapy, photothermal therapy, photodynamic therapy	Therapy	A549 and 3T3 cell lines, A549 tumor-bearing nude mice	[139]
HA-GC-DOX/CXB	Hyaluronic acid	CD44 receptor	pH	DOX, Celecoxib	Chemotherapy	Therapy	A549-Luc cell line, Murine A549-Luc xenograft model	[140]
sp-PA	Biotin	Biotin receptor	Photo, ROS	PTX, silicon 2,3-naphthalocyanine bis	Chemotherapy, photodynamic therapy	Therapy	A549 cell line, Xenograft tumor mouse model	[141]
CS-nanogels	Chondroitin sulfate	CD44 receptor	pH, thermo	Cisplatin	Chemotherapy	Therapy	A549 cell line, A549 tumor-bearing nude mice model	[142]
CCG-NP	Concanavalin A	Mannose receptor	MMP2	Cisplatin	Chemotherapy	Therapy	A549 cell line	[143]
Tf-SS-Afa-LPNs	Transferrin	Transferrin receptor	Redox	Afatinib	Chemotherapy	Therapy	H1975, and PC-9 cell lines, lung cancer-bearing mice	[144]
UA@M-CS-FA	Folic acid	Folate receptor	pH	Ursolic acid	Chemotherapy	Therapy	HeLa and HepG2 cell lines, HeLa bearing tumor model	[145]
FA-BSA@DA	Folic acid	Folate receptor	ROS	Aspirin prodrug	Chemotherapy	Therapy and imaging	A549, MCF-7, and MCF-7 cell lines, breast cancer xenograft tumor models with lung metastasis	[146]
MDNP	Folic acid	Folate receptor	pH, photo	Gemcitabine, NU7441	Chemotherapy, radiotherapy	Therapy	A549 and H460 lung cancer cell lines, H460 tumor-bearing athymic nude mice	[147]
FA/Tf-CDDP-NPs	Folic acid, transferrin	Folate receptor	GSH	Cisplatin	Chemotherapy	Therapy	A549 cell line, A549 tumor-bearing nude mice model	[148]
TBP@DOX	Fucoidan	P-selectin	Microwave	DOX	Chemotherapy	Therapy	HepG2 and H22 cell lines, HepG2 and H22 xenograft tumor models with lung metastasis	[149]
GEM-FGONS	Fucose	Fucose receptor	pH	Gemcitabine hydrochloride	Chemotherapy	Therapy	MDA-MB-231 and A549 cell lines	[150]
PSO-HGNPs-DOX	Polyoxyethylene sorbitol oleate	Low-density lipoprotein receptor	Photo, thermo	DOX	Chemotherapy, radiotherapy, photothermal therapy	Therapy	A549 cell line, A549 human lung cancer-bearing mouse model	[151]
Apt@DP-DOX-MCN	MUC1 aptamer	Cancer cell	pH, GSH	DOX	Chemotherapy	Therapy	A549 and MCF-7 cell lines	[152]
Apt-Fe3O4@C@DOX	sgc8 aptamer	Cancer cell	pH, thermo	DOX	Chemotherapy, photothermal therapy	Therapy and imaging	A549 cell line, A549 tumor-bearing nude mice model	[153]
Au-siRNA-PAA-AS1411	AS1411 aptamer	Nucleolin receptors	Photo, MMP2, magnetic field	DOX, siRNA	Chemotherapy, gene therapy, photothermal therapy	Therapy	NCI-H889 cell line, lung cancer orthotopic murine model	[154]
CDGM	MMP2 peptide	MMP2	pH, photo	DOX, Ce6	Chemotherapy, photodynamic therapy	Therapy and imaging	A549 cell line, A549 tumor-bearing nude mice model	[133]
PEG-SS-Ce6-MMP2	MMP2 peptide	MMP2	Photo, redox	Ce6	Photodynamic therapy	Therapy and imaging	A549 cell line, A549 tumor-bearing nude mice model	[132]
GNS@BSA/I-MMP2	MMP2 peptide	MMP2	Photo	IR 780 iodide	Photodynamic therapy, photothermal therapy	Therapy and imaging	A549 cell line, A549 tumor-bearing nude mice model	[131]
GNPs@PEG/Ce6-P	PD-L1 peptide	PD-L1	Photo	Ce6	Photodynamic therapy	Therapy and imaging	HCC827 cell line, HCC827 tumor-bearing mice	[134]
cNGQ-PS-Dox	cNGQ peptide	α3β1 integrin receptor	Redox	DOX	Chemotherapy	Therapy	A549 cell line, A549 human lung cancer-bearing nude mice model	[155]
cPCPL/siRNA/ETP	cRGDyC peptide	-	pH, redox	EZH2 siRNA—Etoposide	Chemotherapy, gene therapy	Therapy	luc-A549 cell line, nude mice bearing orthotopic NSCLC	[156]
cNGQ-PS-DTX	cNGQGEQc peptide	α3β1 integrin receptor	Redox	DTX	Chemotherapy	Therapy	A549 cell line, orthotopic A549 xenografts	[63]
siBec1@PPN	cRGD peptide	α3β1 integrin receptor	GSH	Cisplatin—Beclin1 siRNA	Chemotherapy, gene Therapy	Therapy	A549 cell line, Cisplatin-resistant A549 tumor on xenograft mice models	[157]
RGD-ss-PTX/CDDP LPNs	RGD peptide	αvβ3 integrin	Redox	PTX, Cisplatin	Chemotherapy	Therapy	A549 and NCI-H1299 cell lines, A549 tumor-bearing mice	[158]
U11-DOX/CUR	U11 peptide	Urokinase plasminogen activator receptor	pH	DOX, Curcumin	Chemotherapy	Therapy	A549/ADR cells line, mice bearing A549/ADR cells cancer model	[159]
T7-DSNPs/9291	T7 peptide	Transferrin receptor	GSH	Osimertinib, DOX	Chemotherapy	Therapy	PC-9 cell line, PC-9 tumor nude mice	[160]

**Table 4:** Clinical trials performed with nanotherapeutics in lung cancer patients.

Trade Name	Particle Type	Therapeutic Agent	Indication	NCT Number	Phase	Status
Abraxane	Albumin based nanoparticle	Paclitaxel	Non-squamous NSCLC	NCT02264990	Phase III	Completed
				NCT03169335	Phase III	Completed
			NSCLC	NCT03594747	Phase III	Ongoing
				NCT03296163	Phase III	Completed
				NCT02754882	Phase III	Completed
BIND-014	Polymeric	DTX	Advanced or metastatic cancer, including lung cancer	NCT01300533	Phase I	Completed
			NSCLC	NCT02283320	Phase II	Completed
				NCT01792479	Phase II	Completed
EP0057 (CRLX101)	Polymeric	Camptothecin	NSCLC	NCT01380769	Phase II	Completed
			Lung Neoplasms, Small Cell Lung Cancer	NCT02769962	Phae I/II	Ongoing
Genexol-PM	Polymeric micelle	Paclitaxel	NSCLC	NCT01770795	Phase II	Completed
				NCT01023347	Phase II	Completed
LY01610	Liposome	Irinotecan hydrochloride	Small Cell Lung Cancer	NCT04381910	Phase II	Ongoing
NBTXR3	Hafnium Oxide-containing Nanoparticles		NSCLC	NCT03589339	Phase I	Ongoing
			Recurrent NSCLC	NCT04505267	Phase I	Ongoing
NC-6004	Micellar	Cisplatin	Solid tumors	NCT02240238	Phase I/II	Completed
NK012	Polymeric micelle	SN-38	Small cell lung cancer	NCT00951613	Phase II	Completed
NKTR-102	Polymeric	Irinotecan	NSCLC	NCT01773109	Phase II	Completed
			Lung and breast cancer	NCT02312622	Phase II	Completed
			Recurrent small cell lung carcinoma	NCT01876446	Phase II	Completed
Oncoprex (DOTAP:Chol-fus1)	Liposomal	FUS1 gene	NSCLC	NCT00059605	Phase I	Completed
				NCT01455389	Phase I/II	Ongoing
				NCT04486833	Phase I/II	Ongoing
Onivyde	Liposomal	Topotecan	Small cell lung cancer	NCT03088813	Phase II/III	Ongoing
Pm-Pac	Polymeric micellar	Paclitaxel	NSCLC	NCT02667743	Phase III	Completed
SGT-53	Cationic liposome	Wild-type p53 DNA sequence	Neoplasm	NCT00470613	Phase I	Completed
TargomiRs	Targeted minicells	miRNA	NSCLC	NCT02369198	Phase I	Completed
Taxoprexin	DHA-bonded paclitaxel		NSCLC	NCT00243867	Phase III	Completed
TLI	Liposomal	Topotecan	Small cell lung cancer	NCT00765973	Phase I	Completed

## Data Availability

Not applicable.
